# Polyhexamethylene Guanidine Phosphate Damages Tight Junctions and the F-Actin Architecture by Activating Calpain-1 via the P2RX7/Ca^2+^ Signaling Pathway

**DOI:** 10.3390/cells9010059

**Published:** 2019-12-24

**Authors:** Sun Woo Jin, Gi Ho Lee, Hoa Thi Pham, Jae Ho Choi, Hye Gwang Jeong

**Affiliations:** College of Pharmacy, Chungnam National University, Daejeon 34134, Korea; mpassword@cnu.ac.kr (S.W.J.); ghk1900@cnu.ac.kr (G.H.L.); hoapt@cnu.ac.kr (H.T.P.); choijh1@cnu.ac.kr (J.H.C.)

**Keywords:** PHMG-p, tight junctions, calpain, F-actin, P2RX7

## Abstract

Polyhexamethylene guanidine phosphate (PHMG-p), a member of the polymeric guanidine family, has strong antimicrobial activity and may increase the risk of inflammation-associated pulmonary fibrosis. However, the effect of PHMG-p on the barrier function of the bronchial epithelium is unknown. Epithelial barrier functioning is maintained by tight junctions (TJs); damage to these TJs is the major cause of epithelial barrier breakdown during lung inflammation. The present study showed that, in BEAS-2B human bronchial epithelial cells, exposure to PHMG-p reduced the number of TJs and the E-cadherin level and impaired the integrity of the F-actin architecture. Furthermore, exposure to PHMG-p stimulated the calcium-dependent protease calpain-1, which breaks down TJs. However, treatment with the calpain-1 inhibitor, ALLN, reversed the PHMG-p-mediated impairment of TJs and the F-actin architecture. Furthermore, exposure to PHMG-p increased the intracellular Ca^2+^ level via P2X purinoreceptor 7 (P2RX7) and inhibition of P2RX7 abolished the PHMG-p-induced calpain-1 activity and protein degradation and increased the intracellular Ca^2+^ level. Although exposure to PHMG-p increased the extracellular ATP level, hydrolysis of extracellular ATP by apyrase did not influence its detrimental effect on bronchial epithelial cells. These results implicate the impairment of TJs and the F-actin architecture in the pathogenesis of pulmonary diseases.

## 1. Introduction

The respiratory epithelial layer protects the surface of the airways and alveoli by trapping and removing infectious particles and airborne pollutants [1]. It is also implicated in a variety of lung diseases, such as asthma and chronic obstructive pulmonary disease, through regulation of the immune response to inflammatory stimuli and promotion of susceptibility to respiratory distress syndrome and lung damage [2,3]. Destruction of the epithelial barrier is a hallmark of respiratory distress syndrome and is confirmed by the presence of high-molecular-weight serum proteins in bronchoalveolar lavage fluid [4].

The epithelial lining of the respiratory tract consists of a monolayer of polarized epithelial cells linked to their neighbors by tight junctions (TJs), adherens junctions (AJs), desmosomes, and gap junctions [5]. TJs and AJs, the most apically located junctions, are collectively referred to as the apical junctional complex, which plays a key role in the formation and maintenance of the epithelial barrier. TJs and AJs consist of transmembrane and peripheral membrane protein complexes [6,7]. Occludin, claudin, and junction adhesion molecules are transmembrane TJ proteins in epithelial cells; these proteins seal the paracellular space and regulate paracellular transport between epithelial cells [8]. In addition, adaptor proteins in the cytoplasm, such as zonula occludens (ZO) proteins, support TJ structures to form a scaffold between the transmembrane proteins and the actin cytoskeleton [8]. AJs in epithelial cells are composed of E-cadherin and proteins of the nectin family [5,6]. ZO proteins, the most extensively studied component of TJ cytoplasmic plaques, mediate interactions between different types of TJs and/or connect them to the actin cytoskeleton [6,7]. The armadillo-family proteins, β-catenin and p120 catenin, are present in the cytoplasmic plaque of AJs and mediate the interaction of E-cadherin with actin-binding proteins, which link E-cadherin to the actin cytoskeleton [6,7]. Rapid apical junctional complex disassembly is associated with the reorganization of actin filaments by F-actin cytoskeleton-disrupting toxins and inflammatory stimuli [9,10].

Polyhexamethylene guanidine (PHMG) is a guanidine-based polymeric antimicrobial agent [11,12,13]. The major two derivatives of its salt, PHMG phosphate (PHMG-p) and PHMG hydrochloride, are widely used in detergents, paints, and swimming pools [14,15,16]. The main antimicrobial mechanism of guanidine derivatives is the destruction of the plasma membrane through the interaction of the cationic nitrogen with negatively charged cell-surface phospholipids, especially phosphatidylglycerol, the main component of the bacterial membrane [17,18,19,20]. The cytotoxicity of guanidine derivatives is also mediated by the destruction of the plasma membrane [12,13]. However, the effect of PHMG on TJs is unclear. Therefore, we evaluated the effect of PHMG on TJs and the F-actin architecture, and characterized the underlying mechanism.

## 2. Materials and Methods

### 2.1. Chemicals and Reagents

Dulbecco’s modified Eagle’s medium/Ham’s F-12 Nutrient Mixture (DMEM/F-12), fetal bovine serum, and trypsin–ethylenediaminetetraacetic acid were purchased from Welgene (Gyeongsan, South Korea). 3-(4,5-Dimethylthiazol-2-yl)-2,5-diphenyltetrazolium bromide (MTT) was purchased from USB Corporation (Cleveland, OH, USA). The lactate dehydrogenase (LDH) assay kit was purchased from Roche Applied Science (Indianapolis, IN, USA). Fluo-4 NW and Alexa Fluor 488-conjugated secondary antibodies were purchased from Invitrogen (Carlsbad, CA, USA). Antibodies against ZO-1, E-cadherin, occludin, claudin-1, and calpain-1, as well as horseradish peroxidase-conjugated anti-mouse and anti-rabbit IgG, were purchased from Cell Signaling Technology (Beverly, MA, USA). The anti-β-actin antibody was purchased from Santa Cruz Biotechnology (Dallas, TX, USA). The enhanced chemiluminescence system was obtained from BioFact (Daejeon, South Korea), and nitrocellulose membranes were purchased from Amersham Pharmacia Biotech (Uppsala, Sweden). Phalloidin, MG 132, ALLN, thapsigargin, and BAPTA-AM were purchased from Sigma-Aldrich (St. Louis, MO, USA). Suramin and A438079 were obtained from Tocris Bioscience (Minneapolis, MN, USA). The Highly Stable ATP Bioluminescence Assay Kit and Calpain Activity Fluorometric Assay Kit were purchased from BioVision (Milpitas, CA, USA). All other chemicals were of the highest grade commercially available.

### 2.2. Cell Culture

BEAS-2B cells were obtained from the American Type Culture Collection (Bethesda, MD, USA). The cells were grown in DMEM/F-12 supplemented with 10% fetal bovine serum, 100 U/mL penicillin, and 100 μg/mL streptomycin (HyClone, Logan, UT, USA) at 37 °C in a humidified incubator with an atmosphere containing 5% CO_2_.

### 2.3. Cell Viability Assay

Conventional MTT reduction and LDH assays were used to determine the toxicity of PHMG-p to BEAS-2B cells. Cells were seeded in 48-well plates and allowed to attach overnight. On the following day, the cells were treated with PHMG-p for 4–24 h. MTT solution was added, followed by incubation for 30 min, and formazan crystals were solubilized by adding DMSO. The absorbance at 550 nm was measured using a BioTek Synergy HT microplate reader (BioTek Instruments, Winooski, VT, USA). The medium was collected for LDH assays, and the absorbance was measured at 490 nm using a BioTek Synergy HT microplate reader. Cell viability (%) and cytotoxicity (fold-change) were quantified based on the absorbance of treated cells relative to control cells.

### 2.4. Immunofluorescence

Cells were grown on glass coverslips to 60–80% confluence, fixed in 4% paraformaldehyde for 20 min at room temperature, and blocked in 5% serum for 30 min. After five washes with phosphate-buffered saline containing 0.2% Tween, the coverslips were immersed in 0.2% Triton X-100 in phosphate-buffered saline for 10 min. Thereafter, cells were incubated with a primary antibody against ZO-1 (1:100) at 4 °C overnight or with phalloidin for 40 min at room temperature to stain F-actin. After they had been washed, the cells that had been incubated with ZO-1 were subsequently incubated with an Alexa Fluor 488-conjugated secondary antibody for 2 h at room temperature. Nuclei were stained with 4’,6-diamidino-2-phenylindole (1:1000 dilution), and the cells were viewed under an EVOS fluorescence microscope (Life Technologies, Carlsbad, CA, USA).

### 2.5. Determination of Intracellular Ca^2+^ Level

The intracellular Ca^2+^ level was measured by Fluo-4 NW staining, in accordance with the manufacturer’s instructions. Briefly, cells were transferred to black 96-well plates, incubated with Fluo-4 NW for 30 min at 37 °C and subsequently in the dark for 30 min at 25 °C. The cells were then treated with PHMG-p, and the intracellular Ca^2+^ concentration was determined at excitation/emission wavelengths of 488/512 nm at 20 s intervals for 5 min using a BioTek Synergy HT microplate reader. Fluorescence images of selected cells were captured using an EVOS fluorescence microscope.

### 2.6. Measurement of Calpain Activity

Calpain activity was evaluated using the Calpain Activity Fluorometric Assay Kit, in accordance with the manufacturer’s instructions. Briefly, cells were lysed in extraction buffer for 30 min on ice and centrifuged at 10,000 rpm for 1 min. Supernatants were collected and protein concentrations were determined using a protein assay kit (Pro-Measure, Intron Biotechnology, Seongnam, South Korea). Supernatants were incubated with substrate (Ac-LLY-AFC) and reaction buffer for 1 h at 37 °C in the dark. Upon cleavage of substrate, the fluorogenic portion was measured at excitation/emission wavelengths of 400/505 nm using a BioTek Synergy HT microplate reader. The results are expressed as relative fold changes from the control group. Additional reactions were performed to compare the control group with 1 μg Active Calpain I (BioVision) to the PHMG-p treated group with 20 μM Z-LLY-FMK (BioVision), a calpain inhibitor. The results are expressed as relative fold changes in fluorescence units.

### 2.7. ATP Assay

The ATP concentration was measured using the Highly Stable ATP Bioluminescence Assay Kit (Biovision). Briefly, cells were treated with PHMG-p; then, 90 μL of luciferin–luciferase assay solution was distributed in the wells of a white 96-well plate and medium (10 μL) was added to each well. Luminescence was recorded using a Varioskan luminometer (Thermo Electron, Waltham, MA, USA). The ATP concentration (μM) was calculated using a standard curve.

### 2.8. Western Blotting

Cell lysates were prepared in lysis buffer (120 mM NaCl, 40 mM Tris [pH 8], and 0.1% Nonidet P-40) on ice for 30 min and centrifuged at 10,000 rpm for 15 min. Supernatants were collected and concentrations were determined at 595 nm using a protein assay kit. Equal amounts of total cellular protein were electrophoresed in 8–15% sodium dodecyl sulfate-polyacrylamide gel electrophoresis gels and transferred to nitrocellulose membranes. After membranes had been blocked in 5% skim milk for 1 h, they were incubated with primary antibodies overnight, then with the appropriate horseradish peroxidase-conjugated secondary antibodies. Protein bands were detected using an enhanced chemiluminescence Western blotting detection kit (Biofact). The integrated optical density for the protein band was calculated by Image-J software version 1.42q (NIH, Bethesda, MD, USA), and then the values were normalized to β-actin level.

### 2.9. Statistical Analysis

All experiments were repeated at least three times. Results are reported as means ± standard deviations. One-way analysis of variance was used to determine significant differences between treatment groups. The Newman–Keuls test was used for comparisons of three or more groups. *p*-values < 0.01 were considered statistically significant.

## 3. Results

### 3.1. Effect of PHMG-p on TJ Proteins and the F-Actin Architecture in BEAS-2B Cells

**Figure 1:**
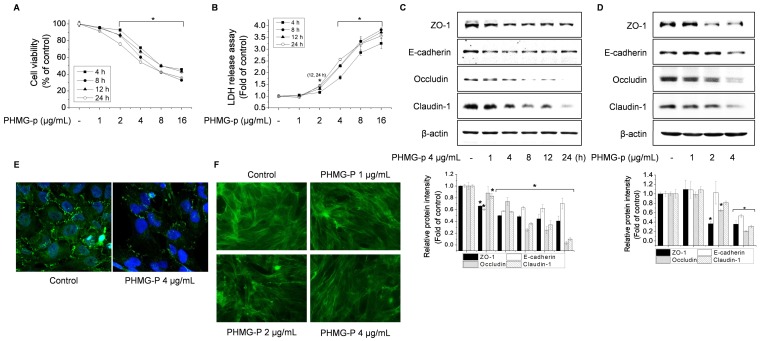
Polyhexamethylene guanidine phosphate (PHMG-p) disrupts the epithelial barrier by degrading tight junction (TJ) proteins and impairing the F-actin architecture in BEAS-2B cells. (**A**,**B**) Cells were treated with 1–16 μg/mL PHMG-p for 4–24 h. Cytotoxicity was assessed by MTT (A) and LDH (**B**) assays. Cells were treated with 4 μg/mL PHMG-p for 1–24 h (**C**) or 1–4 μg/mL PHMG-p for 4 h (**D**–**F**), then assessed by western blotting (**C**,**D**), immunofluorescence (**E**), or F-actin staining (**F**). *p < 0.01, significantly different from the control.

Initially, we determined the concentration range of PHMG-p suitable for BEAS-2B cells by using MTT and LDH assays. The cytotoxicity of PHMG-p increased in a time- and concentration-dependent manner (Figure 1A,B). Exposure to PHMG-p at > 4 μg/mL reduced cell viability by > 50% and increased LDH release threefold. Thus, 4 μg/mL was the maximum PHMG-p concentration used in this study. We evaluated the expression levels of TJ markers (ZO-1, occludin, and claudin-1) and of an AJ marker (E-cadherin), which also contributed to the epithelial barrier function [5,6]. Western blotting showed that exposure to PHMG-p reduced the levels of TJ and AJ proteins in a time- and concentration-dependent manner (Figure 1C,D). Furthermore, immunofluorescence and F-actin staining showed that exposure to PHMG-p altered ZO-1 localization near the cell membrane and reduced the integrity of the F-actin architecture (Figure 1E,F).

### 3.2. Role of Calpain in Impairment of TJ Proteins and F-Actin Architecture by PHMG-p

**Figure 2:**
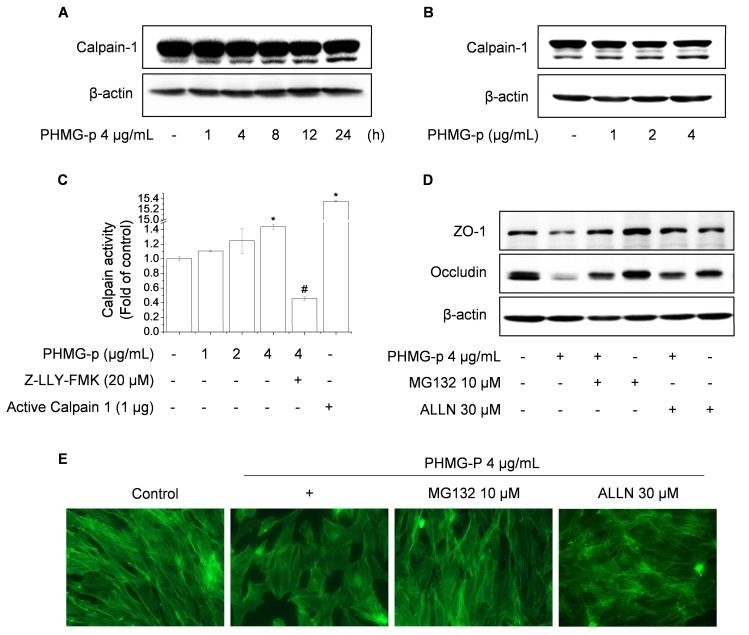
Activated calpain-1 is required for PHMG-p-mediated degradation of tight junctions. Cells were treated with 4 μg/mL PHMG-p for 1–24 h (**A**) or 1–4 μg/mL PHMG-p for 4 h (**B**), then assessed by western blotting. (C) Cells were treated with 1–4 μg/mL with or without active calpain-1 (positive control) and Z-LLY-FMK (calpain inhibitor), then subjected to calpain activity assay. (**D**,**E**) Cells were treated with 10 μM MG132 or 30 μM ALLN, followed by incubation with 4 μg/mL PHMG-p for 4 h, then assessed by western blotting (**D**) or F-actin staining (**E**). *p < 0.01, significantly different from the control. #*p* < 0.01, significantly different from PHMG-p-treated cells.

Intracellular proteases are activated during inflammation and cancer, promoting intracellular cleavage of junction proteins. Sumitomo et al. (2011) demonstrated that streptolysin S from Group A Streptococcus induces calpain activity, leading to the cleavage of occludin and impairment of the barrier function of keratinocytes and intestinal epithelial cells [21]. Wang et al. (2012) showed that activated calpain, following exposure to particulate matter, mediated ZO-1 degradation in human lung microvascular epithelial cells [22]. Accordingly, we investigated the role of calpain in PHMG-p-mediated degradation of TJ proteins. Exposure to PHMG-p increased the level of active calpain-1 and the proteolytic activity of calpain (Figure 2A–C). Furthermore, treatment with the proteasome inhibitor, MG-132, and the calpain-1 inhibitor, ALLN, abolished the PHMG-p-mediated degradation of TJs and the altered F-actin architecture (Figure 2D,E). In addition, Figure S1 showed that ALLN significantly suppressed PHMG-p-reduced cell viability. Taken together, PHMG-p-mediated calpain-1 activation is key event to lead the reduction of cell viability as well as the impairment of tight junctions and the F-actin architecture.

### 3.3. PHMG-p Induces Intracellular Ca^2+^ Influx via P2RX7

**Figure 3:**
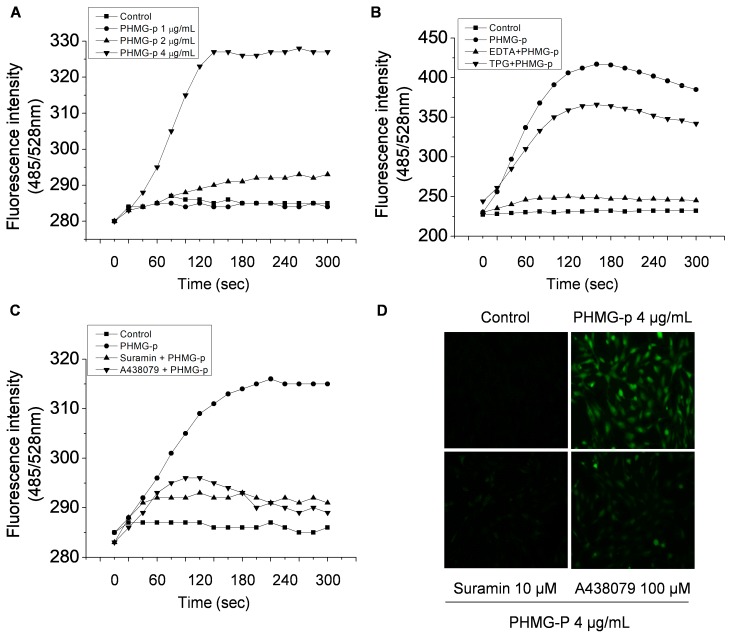
PHMG-p induces intracellular Ca^2+^ influx via P2RX7. Cells were treated with 1–4 μg/mL PHMG-p (**A**); 0.5 mM ethylenediaminetetraacetic acid and 1 μM thapsigargin, followed by incubation with 4 μg/mL PHMG-p (**B**); or 10 μM suramin and 100 μM A438079, followed by 4 μg/mL PHMG-p (**C**,**D**). Cells were assessed by Fluo-4 NW staining at 20-s intervals for 5 min (**A**–**C**) and visualized by fluorescence microscopy (**D**).

Calpains are calcium-activated cysteine proteases that exist as various isoforms (e.g., μ- and m-calpain or calpain-1 and -2). We investigated whether exposure to PHMG-p induces intracellular Ca^2+^ influx. Indeed, PHMG-p induced intracellular Ca2+ influx, which could be blocked by using ethylenediaminetetraacetic acid (Figure 3A,B). However, thapsigargin, an inhibitor of sarco/endoplasmic reticulum Ca^2+^-ATPase, did not affect the PHMG-p-induced increase in the intracellular Ca^2+^ level (Figure 3B). These results suggest that the source of Ca^2+^ is extracellular.

The P2RX7 receptor, a member of the purinergic type-2 receptor family, is a ligand-gated ion channel. P2RX7 signaling plays an important role in Ca^2+^-related signaling pathways in the epithelium of the airways and alveoli [23]. Song et al. (2016) reported that several purinergic receptors are expressed in BEAS-2B cells [24]. Therefore, we investigated whether P2RX7 is involved in intracellular Ca^2+^ influx and calpain 1 activation. A general inhibitor of purinergic receptors, suramin, and a P2RX7 inhibitor, A438079, reduced the PHMG-p-induced increase in the intracellular Ca^2+^ level (Figure 3C and D). These results suggested that P2RX7 is a Ca^2+^ channel targeted by P2RX7.

### 3.4. PHMG-p Induces Calpain-1 Activity and Calpain-1-Dependent Epithelial Barrier Dysfunction via P2RX7/Ca^2+^

**Figure 4:**
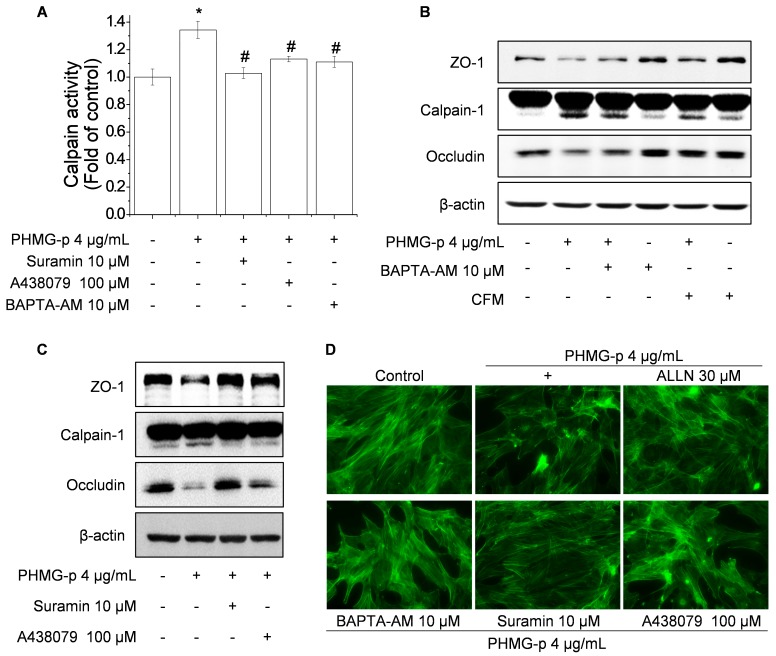
Blockade of PHMG-p-mediated intracellular Ca^2+^ influx reduces calpain-1 activity and calpain-1-dependent disruption of the epithelial barrier. (**A**) Cells were treated with or without 10 μM suramin, 100 μM A438079, and 10 μM BAPTA-AM, followed by incubation with 4 μg/mL PHMG-p, then subjected to calpain activity assay. (**B**,**C**) Cells were treated with 10 μM BAPTA-AM, 10 μM suramin, and 100 μM A438079, or medium was replaced with Ca^2+^-free DMEM/F-12 (CFM), followed by incubation with 4 μg/mL PHMG-p and western blotting. (**D**) Cells were treated with or without 10 μM suramin, 100 μM A438079, 10 μM BAPTA-AM, and 30 μM ALLN, followed by incubation with 4 μg/mL PHMG-p and F-actin staining. *p < 0.01, significantly different from the control. #*p* < 0.01, significantly different from PHMG-p-treated cells.

To determine whether PHMG-induced intracellular Ca^2+^ influx affects calpain-1 activation, we investigated the effect of P2RX7 blockade on calpain-1 activity. Treatment with suramin, A438079, and BAPTA-AM suppressed the PHMG-induced activity of calpain (Figure 4A) and the active form calpain-1 (Figure 4B,C). Furthermore, blockade of Ca^2+^ channels and depletion of Ca^2+^ reversed the PHMG-p-mediated degradation of TJs (Figure 4B,C) and altered F-actin architecture (Figure 4D). P2RX7 is an ATP-gated cation channel, and its activation by extracellular ATP (eATP) results in a variety of cellular responses, including Ca^2+^ influx, membrane pore formation, and cytokine production [25]. Accordingly, we investigated whether PHMG-p induces intracellular Ca^2+^ influx by P2RX7 through an increase the eATP level. PHMG-p increased the eATP level (Figure S2A); however, the hydrolysis of eATP by apyrase did not affect PHMG-p-mediated intracellular Ca^2+^ influx and degradation of TJs (Figure S2B,C). Therefore, the P2RX7-mediated intracellular Ca^2+^ is required for calpain-1 activation by PHMG-p, whereas P2RX7 activation by PHMG-p is not involved in the increased eATP level.

## 4. Discussion

We investigated the molecular mechanism by which PHMG-p promotes disruption of the epithelial barrier in BEAS-2B cells. One of our principal findings was that PHMG-p induced the degradation of TJs and impairment of F-actin integrity by activation of calpain-1. Furthermore, PHMG-p increased calpain-1 activity via the promotion of intracellular Ca2 influx through P2RX7, which was essential for its effect on TJ proteins and the F-actin architecture.

TJs in the lung epithelium play a key role in barrier function and form the sealing interface between adjacent epithelial cells [26]. During lung inflammation, the destruction of TJ proteins disrupts the epithelial barrier. In this study, we found that PHMG-p reduced the levels of TJ markers (ZO-1, occludin, and claudin-1) and of an AJ marker (E-cadherin) in BEAS-2B cells. Similarly, PHMG-p has been reported to reduce transepithelial electrical resistance in a bronchial ALI co-culture model; notably, transepithelial electrical resistance is involved in the disruption of the airway epithelial barrier [27]. Song et al. (2019) reported that the cationic nature of PHMG-p could disrupt membrane pore formation or integrity in lung epithelial cells, fibroblasts, and monocytes [13]. Furthermore, F-actin cytoskeleton-disrupting toxins have been shown to induce rapid AJ/TJ disassembly, indicating that the reorganization of actin filaments is responsible for the loss of AJs during inflammation [9]. Inflammatory stimuli reportedly induce F-actin alterations and AJ/TJ disassembly [10]. In this study, exposure to PHMG-p altered F-actin integrity in a concentration-dependent manner, consistent with degradation of apical junctional complex proteins. Therefore, we concluded that PHMG-p may disrupt the epithelial barrier by impairing TJs and the F-actin architecture in pulmonary epithelial cells.

TJ proteins are associated with the underlying actin cytoskeleton through a scaffold protein that constrains their intracellular domains, which can also be targeted by proteases located near the cytoplasmic surface of the plasma membrane [28]. Intracellular and membrane-related proteases, such as calpain, mediate the intracellular cleavage of junction proteins, thereby promoting cytoskeletal alteration [29]. Wang et al. (2012) showed that particulate-matter air pollution could induce calpain-mediated degradation of TJ proteins and disrupt the endothelial barrier [22]. Heijink et al. (2012) reported that cigarette smoke extract reduces epithelial integrity by epidermal growth factor receptor- and calpain-dependent disruption of intercellular contacts, which may increase susceptibility to environmental insults such as inhaled pathogens [30]. In this study, exposure to PHMG-p was observed to activate calpain-1 and increase its activity. In addition, the calpain-1 inhibitor ALLN suppressed PHMG-p-mediated degradation of TJs and alteration of F-actin structure. These results suggest that PHMG-p impairs TJ proteins and F-actin in a calpain-1-dependent manner. Interestingly, inhibition of proteasome activity by MG-132 showed the similar effect of ALLN on PHMG-p-mediated disruption of the epithelial barrier, but further studies are needed to determine how PHMG-p affect degradation of TJs and alteration of F-actin structure via proteasome activity.

P2RX7 receptor hyperactivation is implicated in the pathogenesis of a variety of respiratory diseases, including pulmonary fibrosis, acute lung injury, asthma, pulmonary hypertension, and chronic obstructive pulmonary disease [31,32]. Treatment with A438079, a highly selective antagonist of P2RX7, attenuated hyperpermeability in a model of the human blood–brain barrier and suppressed the ZO-1 and occludin levels [33]. In addition, Wesslau et al. (2019) reported a robust increase in epithelial junction adhesion molecule-A expression in P2RX7-knockout mice, compared to wild-type mice; moreover, they reported that inhibition of P2RX7 by oxATP increased the junction adhesion molecule-A protein level [34]. In the present study, we found that exposure to PHMG-p induced intracellular Ca^2+^ influx via P2RX7; furthermore, the inhibition of P2RX7 by A438079 suppressed the PHMG-p-mediated degradation of TJs and altered F-actin structure by reducing Ca^2+^-dependent calpain-1 activity. The P2RX7 signaling pathway is involved in NLRP3 inflammasome activation, and our data are consistent with the findings of a previous report, in which PHMG-p induced lung fibrosis and pulmonary inflammation through activation of the NLRP3 inflammasome in mouse lung tissue [35]. Although ATP is a selective endogenous ligand of P2RX7, and it has low affinity for ATP; thus, high levels of eATP (in the millimolar range) are required for P2RX7-dependent cellular responses in vitro [25,36]. In the present study, exposure to PHMG-p in the nanomolar range increased the eATP level, while eATP hydrolysis by apyrase did not affect PHMG-p-mediated intracellular Ca^2+^ influx, calpain-1 activation, or TJ degradation. P2RX7 can be activated by structurally unrelated agents, such as the antibiotic, polymyxin B [37]; the bactericidal peptide, LL-37 [38]; an amyloidogenic β peptide [39]; and serum amyloid A [40]. Other agents, such as Alu-RNA, have been suggested to activate P2RX7 by acting on the intracellular N- or C-terminal domains [41]. Therefore, the PHMG-p-induced increase in eATP might not be involved in the activation of P2RX7.

## 5. Conclusions

**Figure 5:**
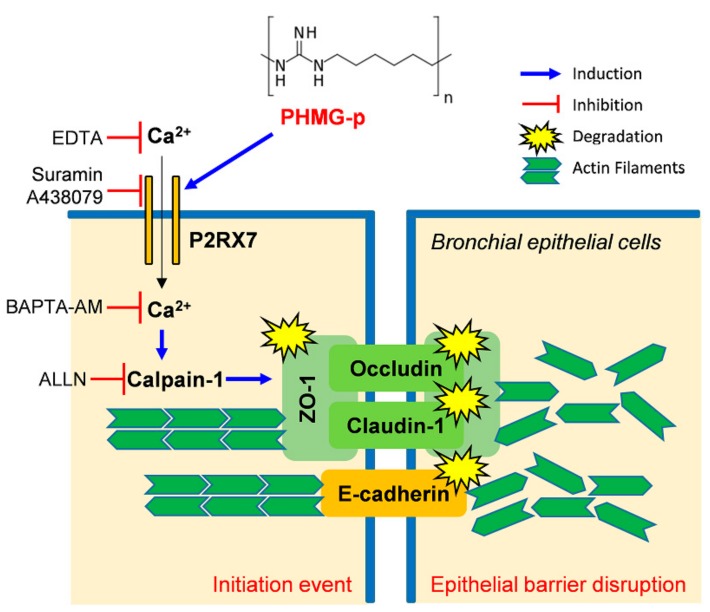
Schematic diagram illustrating the mechanism by which PHMG-p induced the degradation of TJ proteins and impairment of F-actin structural integrity by stimulating the proteolytic activity of calpain-1 via the P2RX7/Ca2+ signaling pathway.

In conclusion, exposure to PHMG-p induced the degradation of TJ proteins and impairment of F-actin structural integrity by stimulating the proteolytic activity of calpain-1. PHMG-p-mediated calpain-1 activity promoted intracellular Ca^2+^ influx via ATP-independent activation of P2RX7. These results suggest that PHMG-p-mediated activation of calpain-1 via the P2RX7/Ca^2+^ signaling pathway could induce pulmonary disease by impairing the barrier function of the bronchial epithelium (Figure 5).

## Supplementary Materials

The following are available online at https://www.mdpi.com/2073-4409/9/1/59/s1, Figure S1: PHMG-p decreases cell viability by calapin-1 activation; Figure S2: PHMG-p induces ATP-independent activation of P2RX7.

## Abbreviations

AJs	adherens junctions
DMEM/F-12	Dulbecco’s modified Eagle’s medium/Ham’s F-12 Nutrient Mixture
eATP	extracellular ATP
LDH	lactate dehydrogenase
MTT	3-(4,5-Dimethylthiazol-2-yl)-2,5-diphenyltetrazolium bromide
PHMG-p	polyhexamethylene guanidine phosphate
P2RX7	P2X purinoreceptor 7
TJs	tight junctions
ZO	zonula occludens
