# Supplementary material for: Polyhexamethylene Guanidine Phosphate Damages Tight Junctions and the F-Actin Architecture by Activating Calpain-1 via the P2RX7/Ca2+ Signaling Pathway
Source: Cells. 2019 Dec 24;9(1):59. doi: 10.3390/cells9010059 (PMC7016582; doi:10.3390/cells9010059)
Supplement: Supplementary file 1 [file cells-09-00059-s001.pdf]

Supplementary Figure

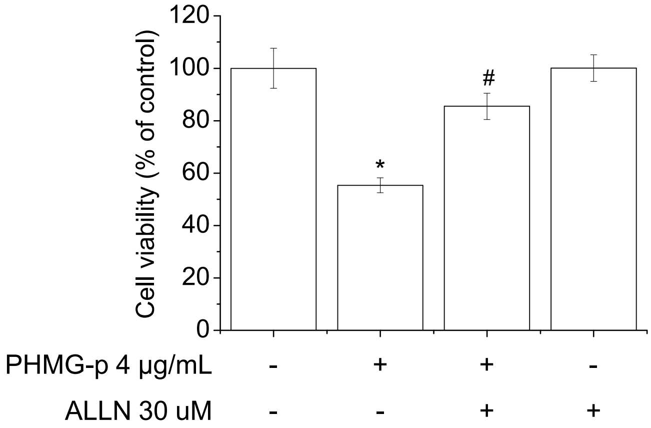

Figure S1. PHMG-p decreases cell viability by calapin-1 activation. Cells were treated with 30 μM ALLN followed by incubation with 4 μg/mL PHMG-p for 4 h. Cell viability was assessed by MTT assay. *$p < 0.01$, significantly different from the control. #$p < 0.01$, significantly different from PHMG-p-treated cells.

Supplementary Figure

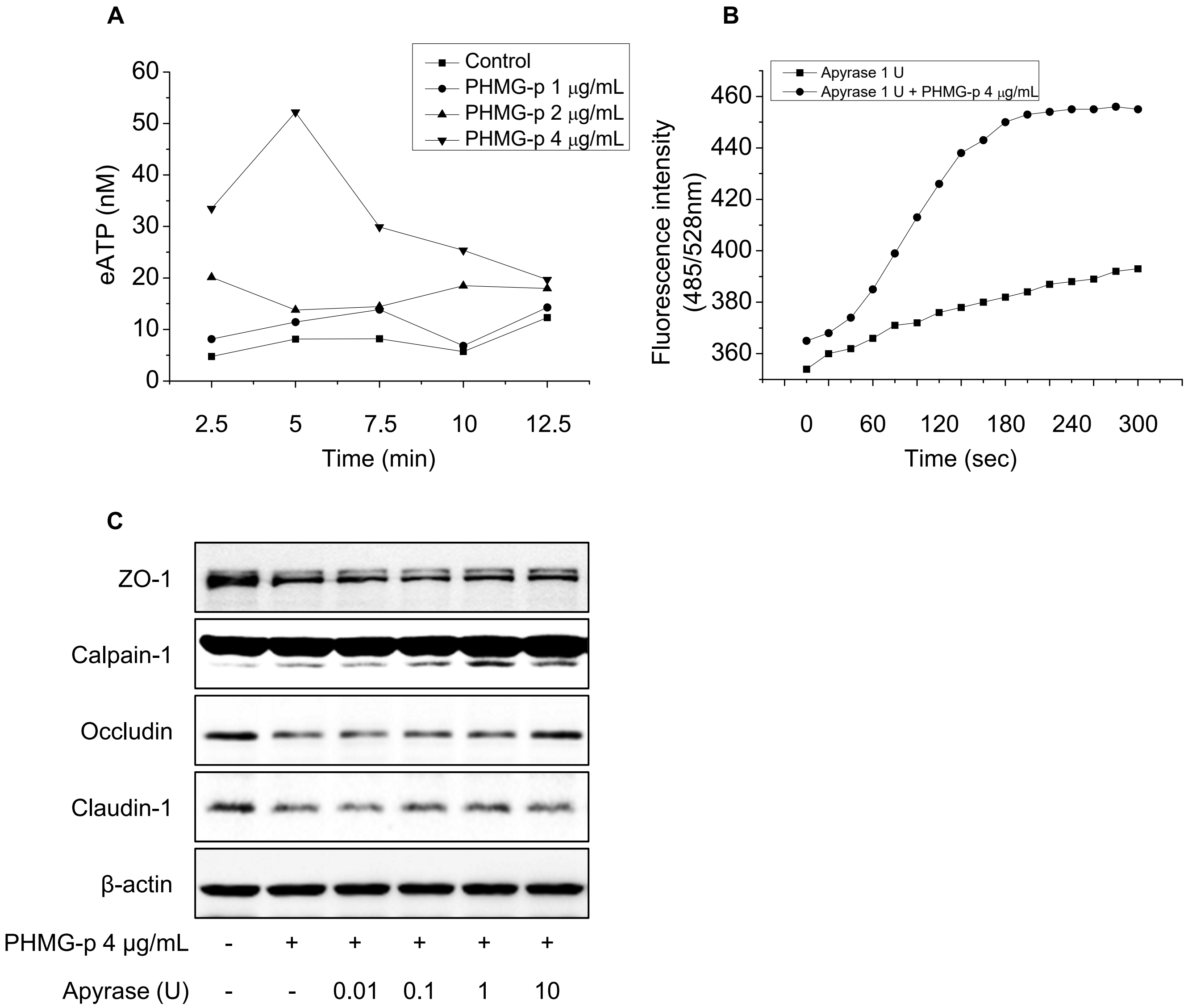

Figure S2. PHMG-p induces ATP-independent activation of P2RX7. (A) Cells were treated with 1-4 μg/ml PHMG-p at 2.5 min interval for 12.5 min and measured by ATP assay. (B) Cells were treated with 1 U apyrase, followed by incubation with 4 μg/ml PHMG-p and assessed by Fluo-4 NW at 20 second intervals for 5 min. (C) Cells were treated with 0.01-10 U apyrase, followed by incubation with 4 μg/ml PHMG-p and assessed by western blotting.
